# Elevated Interleukin-37 Associated with Dengue Viral Load in Patients with Dengue Fever

**DOI:** 10.1007/s00284-023-03239-7

**Published:** 2023-04-06

**Authors:** Jun-Ai Zhang, Jia-Jun Wang, Wen-Ting Zhang, Li Zhang, Bi-Ying Zheng, Gan-Bin Liu, Jing Liang, Yuan-Bin Lu, Xian-Jin Wu, Shu-Ying Yao, Guo-Ying Chen, Yun-Qi Xie, Jun-Yi Wu, Jia-Hua Shi, Jiang Pi, Si-Ping Li, Jun-Fa Xu

**Affiliations:** 1 Guangdong Provincial Key Laboratory of Medical Molecular Diagnostics, The First Dongguan Affiliated Hospital, Guangdong Medical University, Dongguan, China; 2 Institute of Laboratory Medicine, School of Medical Technology, Guangdong Medical University, Dongguan, China; 3 Dongguan Eighth People’s Hospital, Dongguan, China; 4 Department of Respiration, Dongguan 6th Hospital, Dongguan, China; 5 Department of Clinical Laboratory, Huizhou Central People’s Hospital, Huizhou, China

## Abstract

Dengue remains a public health issue worldwide. Similar to chronic infectious diseases, stimulation of cytokine production is not enough to drive immune effector cells for effective virus clearance. One possible mechanism is the virus induces a large number of negative stimulatory cytokines inhibiting immune response. Interleukin 37 (IL-37) plays a crucial regulatory role in infection and immunity, inhibits innate and adaptive immunity as an anti-inflammatory cytokine by inhibiting proinflammatory mediators and pathways. To date, there are few studies reporting correlations between dengue fever (DF) and IL-37. In this study we found that the serum IL-37b and IL-37b-producing monocytes in patients were significantly increased in DF patients. A majority of the IL-37b produced by DF patients was produced by monocytes, not lymphocytes. Increased levels of IL-6, IL-10, and IFN-α were also found in DF patients. However, we failed to detect IL-1β, IL-17A and TNF-α in plasma, because of off-target. In our study, there was no relation between IL-6, IL-10, and IFN-α expressions and IL-37b in serum (*P* > 0.05). The IL-37b-producing monocytes were negatively correlated with the level of IFN-α in serum and platelet count, and positively correlated with lymphocytes percentage (*P* < 0.05, respectively). Additionally, serum DENV nonstructural protein 1 levels were positively correlated with monocytes percentages (*P* < 0.05). Our data represents findings for IL-37b expression and its potential mechanisms in DF patients’ immune response.

**Supplementary Information:**

The online version contains supplementary material available at 10.1007/s00284-023-03239-7.

## Introduction

Each year, almost 400 million people worldwide contract dengue, an acute viral febrile disease transmitted mainly by Aedes aegypti. Caused by dengue virus (DENV), it is one of the most widespread vector-borne infectious diseases in the world [1]. Belonging to the Flavivirus family, DENV is a single positive-stranded RNA virus comprised of four different serotypes [2, 3]. Because of the widespread geographical distribution, secondary infections always occur [4]. Parallel to this, secondary infections with a different DENV serotype more easily develops into severe dengue [5].

Clinically, most dengue infected patients exhibit no symptoms or just mild symptoms. But a minority of patients may get characteristic organ lesions and/or the involvement of central nervous system dysfunction. In severe dengue, hemorrhage, edema, encephalopathy, encephalitis and meningitis are commonly seen [5, 6]. Some dengue infected patients may develop dengue hemorrhagic fever (DHF). Levels of inflammatory cytokines, such as IL-6, IL-10, TNF-α, are elevated in patients with DHF due to ‘cytokine storm,’ leading to endothelial permeability and vascular leak [7, 8]. More seriously, a few percent of dengue infected patients may develop dengue shock syndrome (DSS) which is life threatening [3]. Thus, early diagnosis of dengue infection is very important.

DENV nonstructural protein 1 (NS1) is a glycosylated 48-kD protein that plays a significant role in virus infection. High levels of NS1 are always linked to severe disease [9, 10]. Secreted soluble DENV NS1 (sNS1) is presented in the serum of patients during the acute phase and used as a diagnostic marker for DENV infection, similar with viral RNA [9].

In chronic infectious diseases, anti-inflammatory cytokine elevation is not enough to drive the immune effector cells for effective virus clearance. One possible mechanism is the virus may induce a large number of anti-inflammatory cytokines to inhibit the inflammatory effects. In a previous study, we found that IL-37b was particularly sensitive to tuberculosis infection and maintained high secretion levels in tuberculosis patients [11]. Although, there are some studies showing that IL-6, IL-10 and TNF-α are related to dengue, the roles of IL-37b in dengue fever have not been established. Whether and how DF influences IL-37b and other inflammatory cytokines also requires further investigation. To enhance the knowledge and awareness of DF, we found that the serum IL-37b and IL-37b-producing monocytes were significantly increased in DF patients. What’s more, we also evaluated the correlation between IL-37b and other immune factors in DF patients.

## Materials and Methods

### Subjects and Ethics Statement

A total of 53 cases of dengue fever (DF) patients were recruited from The Eighth People's Hospital of Dongguan City (Dongguan, China). Twenty healthy volunteers were used as controls (HCs). All members were from different age categories and genders (Tables 1 and 2). In terms of age and gender, there was no significant differences between patients and HCs. (The analysis was performed by One-way ANOVA). Those with HIV infection, diabetes, cancer, autoimmune diseases, or immunosuppressive treatment were not included in the study. Neither Guangdong Medical University nor The Eighth People's Hospital of Dongguan City's Internal Review Board approved the study, and this study was conducted with the written consent of all participants.

**Table 1:** Statistic information of study objects

Characteristics	DF	HC	P
Age(Years)	43.60 ± 16.95	41.85 ± 11.83	> 0.05
Gender (F/M)	20/33	7/ 13	> 0.05
Dengue NS1( ±)	53/0	0/20	–

**Table 2:** Age distribution of study objects

Age (Years)	≤ 20	21–30	31–40	41–50	51–60	61–70	71–80	81–90
DF	5	10	4	10	19	2	2	1
HC	0	4	6	4	6	0	0	0

### Peripheral Blood Mononuclear Cells (PBMCs) Preparation and Plasma Conservation

PBMCs were isolated by differential centrifugation over Ficoll (LTS1077, Tianjin Hao Yang Biological Manufacture Co., Ltd.). In the first step, patients and controls provided venous blood samples (5 ml) in tubes containing acid citrate dextrose (ACD). In preparation for PBMC isolation, blood samples were centrifuged at 1500 r/min for 5 min at 20 °C, followed by plasma removal. Whole blood cells were diluted with 2–3 ml PBS. Using a 15 ml conical tube pre-filled with 5 ml Ficoll, the diluted blood was decanted into the Lymphoprep tube for centrifugation at 450×*g* for 25 min at 20 °C without braking. As a result of centrifugation, an upper layer of plasma was formed with a cloudy band of PBMCs and a lower layer of erythrocytes and polymorphonuclear cells formed. After centrifugation, a sterile transfer pipette was used to collect the cloudy PBMC band, which was then added to a 15 ml conical tube filled with 10 ml of PBS. We mixed the 15 ml conical tube by inversion and centrifuged it at 250×*g* for 10 min at 4 °C. After removing the supernatant, the cell pellet was washed again. A second centrifugation step was followed by carefully aspirating the supernatant without disturbing the cell pellet, and PBMCs were resuspended in PBS for the following experiments or resuspended in 1 ml of freezing medium composed of FBS and 10% DMSO and stored at − 80 °C for long-term storage.

### Flow Cytometry Analysis (FCM)

With intracellular flow cytometry staining, IL-37b expression was determined in monocytes. Fixation/Permeabilization kit was used to fixed and permeabilized PBMCs. An additional 30 min of staining was performed with a mouse anti-human IL-37b mAb (6A6, LifeSpan BioSciences), followed by 20 min incubation with 0.5 μl goat anti-mouse IgG/IgM-FITC (555988, BD Biosciences). The stained cells are resuspended in PBS (2% paraformaldehyde and 2% FBS). Then we acquired the samples on a BD FACS-Canto II flow cytometer, finally the results were analyzed using FlowJo software (Tree Star).

### Serum Sample Detection

The serum IL-37b, Dengue virus antigen NS1 was assayed with instruction of commercial ELISA kits (AG-45A-0041YEK-KI01, AdipoGen, Switzerland. Sensitivity 10 pg/ml, assay range 0.016 ~ ng/ml; ARG81357, arigobio, Taiwan, China. Sensitivity 0.5 ng/ml, assay range 1.5–100 ng/ml), the TECAN automatic ELISA analyzer was used to analyze the samples. The serum IL-1β, IL-6, IL-10, IL-17A, TNF-α and IFN-α were assayed with Procartaplex Mix&Match 6-plex (85-EPX060-00000-901, eBioscience, USA), the MAGPIX System and an xPONENT 4.2 MAGPIX analyzer (Luminex) were used to analyze the samples.

### Quantitative Real-Time PCR (RT-qPCR)

RT-qPCR was performed to detect the IL-37b mRNA expression in PBMCs from DF patients and HCs. The PBMCs samples were lysed with Trizol reagent (9109, TaKaRa, Japan) and stored at − 80 °C until RNA extraction. Total RNA was extracted using chloroform according to the manufacturer’s instructions. Add 1 ml of Trizol reagent at 1 × 107 cells, or 0.5 ml for less than 1 × 107 cells, and let stand at room temperature for 5 min. Add 1/5 volume of chloroform and shake well for 15 s. After standing on ice for 5 min, centrifuge at 4 °C at 12,000×*g* for 15 min; aspirate the supernatant into a new RNase-Free EP tube, add the same volume of isopropanol as the supernatant, mix upside down 10 times, stand at room temperature for 3 min, then centrifuge at 4 °C at 12,000×*g* for 15 min; remove the supernatant. The supernatant was removed, washed once with 75% ethanol (DEPC water preparation) pre-chilled at 4 °C, centrifuged at 7500×*g* at 4 °C for 5 min; washed once with anhydrous ethanol pre-chilled at 4 °C, centrifuged at 7500×*g* at 4 °C for 5 min, inverted and left at room temperature for 7 min, and after the liquid at the bottom of the tube evaporated, the RNA was resuspended by adding the appropriate amount of DEPC water. The RNA concentration and purity were determined by UV spectrophotometer (UV-1000, Macy, Shanghai, China), and the absorbance A260/280 ratio was in the range of 1.8–2.2, indicating that the RNA quality was qualified. The cDNA was synthesized by reverse transcription using the PrimeScriptTM RT reagent kit (TaKaRa, Japan) according to the instructions of the reverse transcription kit. Configuration of genomic DNA removal reaction solution: 2.0 μl 5× gDNA Eraser Buffer, 1.0 μl gDNA Eraser, 500 ng Total RNA, up to 10.0 μl RNase Free dH_2_O reaction conditions were 42 °C, 2 min; stored at 4 °C. MasterMix configuration: 4.0 μl RNase Free dH_2_O, 4.0 μl 5× PrimeScript Buffer 2, 1.0 μl RT Primer Mix, 1.0 μl PrimeScript RT Enzyme Mix I. MasterMix was mixed well and 10 µl was added to each tube after the genomic DNA reaction, gently mixed and immediately put into the machine for reverse transcription reaction. The reverse transcription reaction conditions were: 37 °C for 20 min, 85 °C for 5 s, and stored at 4 °C. The cDNA products were diluted 10 times and used for real-time qPCR. The DNA concentration and purity were determined by UV spectrophotometer (UV-1000, Macy, Shanghai, China). Subsequently, RT-qPCR was accomplished using TB Green® Premix Ex Taq™ II (Tli RNaseH Plus) (TaKaRa, Japan) on a QuantStudio™ 6 Flex Real-Time PCR System (Applied Biosystems, Foster City, CA, USA). Primer sequences of were listed as below: human IL-37b: forward, 5′-GTGCTGCTTAGAAGACCCG-3′, reverse, 5′-ATGAGATTCCCAGAGTCCAG-3′; human β-actin: forward, 5′-GGTCGGAGTCAACGGATTTG-3′, reverse, 5′-ATGAGCCCCAGCCTTCTCCAT-3′. The primer sequences were verified by Blast to be correct and specific.

### Single cell RNA-seq (scRNA-seq)

scRNA-seq dataset was downloaded from the NIH GEO database (GSE154386). R package Seurat (v4.0.5) was used for multi-sample integration, data normalization, dimensional reduction, visualization, and differential gene expression analysis [12, 13]. The dataset was filtered to only contain cells with between 2000 and 6000 unique features, 1000–20,000 RNA counts and < 10% mitochondrial RNA content to remove possible doublets. To eliminate erythrocyte contamination, datasets were additionally filtered to contain cells with less than a 5% erythrocytic gene signature (defined as HBA1, HBA2, HBB. Following that SCTransfrom(), SelectIntegrationFeatures() and PrepSCTIntegration() functions were applied to corrected data. PCA was run by 3000 variable features. The first 20 resultant PCs were used to perform a TSNE dimensional reduction in the dataset RunTSEN(). Finally, FindNeighbors() function was used to construct a nearest neighbor graph and FindClusters() function was applied to cluster the dataset with resolution set to 0.4 [13].

### Statistical Analysis

Normality test was first performed to determine if the data approximated a normal distribution. Student’s t-test or ANOVA was employed to compare the differences in measured data by R package ggplot2 [14], and the degree of dependency between variables was measure by Spearman´s correlation analysis through R package ggpubr. Data were shown as Mean ± SEM. P < 0.05 was considered as statistically significant.

## Results

### scRNA-seq Shows Inflammatory Cytokines in DF Patients are Enriched in Monocytes

**Fig. 1:**
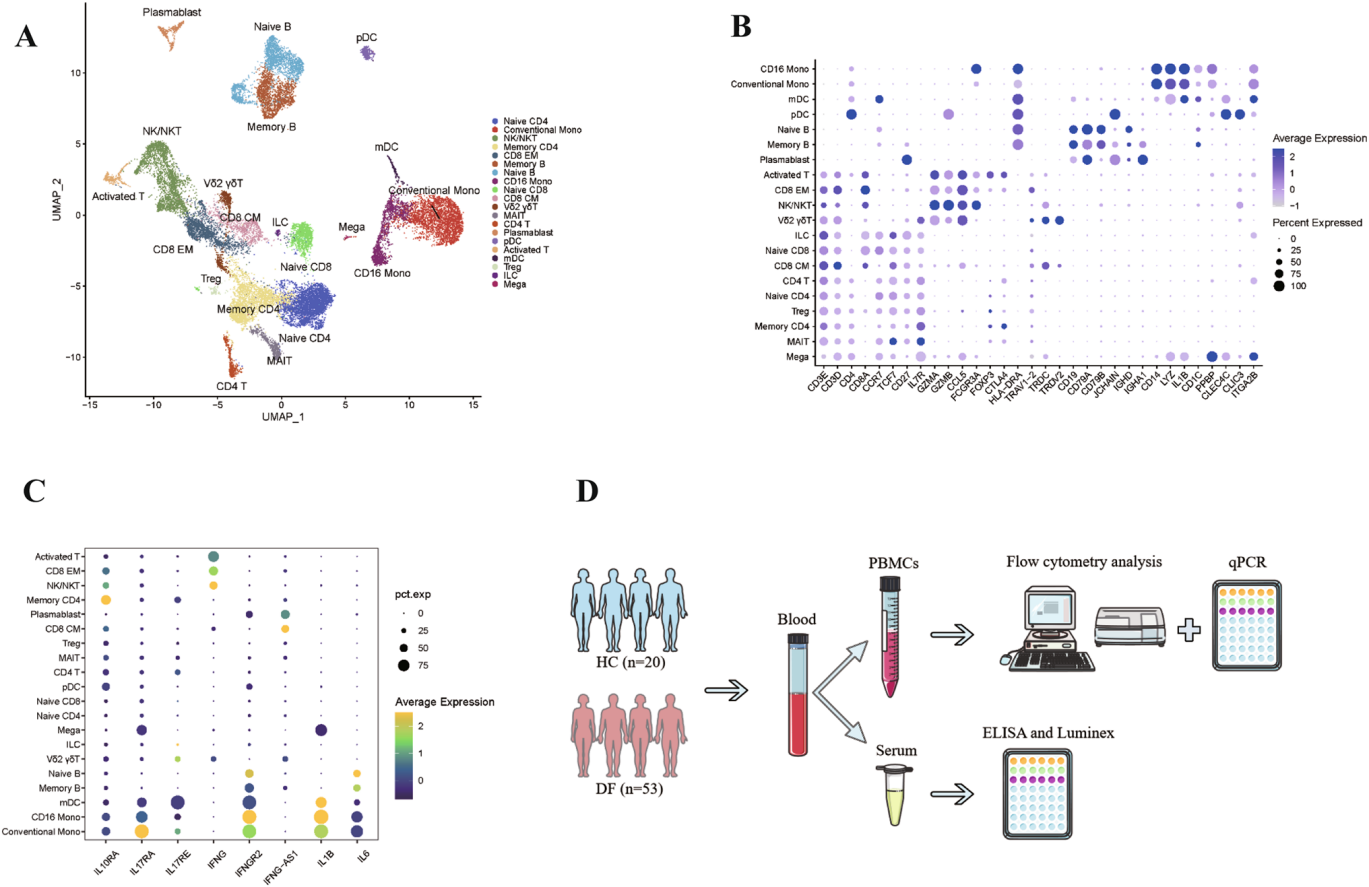
Single cell sequencing data from a publicly available single-cell RNA seq dataset from the NIH GEO database (GSE154386) [13]. **A** Integrated UMAP projection of scRNA-seq data. **B** Expression of key linage defining gene products across all annotated leukocyte populations. **C** Distribution of inflammatory cytokines IL10RA, IL17RA, IL17RE, IFNGR2, IL1B, and IL6. **D** Schematic diagram of the experimental design and analysis

Firstly, a publicly available single-cell RNA seq dataset from the NIH GEO database (GSE154386) were re-analyzed [13]. We extracted the original author's sequencing dataset of natural DENV-1 infection (rather than experimental primary DENV-1 infection), and used dataset of day 180 as control. Using the original author's Maker gene as the clustering basis, cells were divided into 20 cell types by integrated UMAP projection of scRNA-seq (Fig. 1A, B). Then analyzed the distribution of the related inflammatory cytokines with differential expression (*P* < 0.05). We found that IL10RA, IL17RA, IL17RE, IFNGR2, IL1B, and IL6 were mainly distributed in monocytes and monocytes-derived dendritic cells (mDCs) (Fig. 1C). Among them, IL-1β, IL-6, IL-17A are all strong pro-inflammatory factors and increase after DENV infection [15–18], it can be seen that after DENV infection, the inflammatory effect of monocytes is significantly enhanced, in contrast, IL-10 plays a dual role, i.e., it is both pro-inflammatory and anti-inflammatory [19]. As reported, in primary DF, IL-10 serum protein levels were significantly elevated [20], Patients with DHF/DSS showed higher levels of IL-10 than patients with DF, and IL-10 may limit the immunopathology [21]. These suggested that IL-10 acts as an anti-inflammatory factor to antagonize DENV infection to a certain extent. Therefore, we proposed that in DENV infection, in monocytes with the most obvious changes in effector factors, there should be other anti-inflammatory factors to resist the inflammatory response in addition to IL10, such as IL-37, whose anti-inflammatory effect has been widely verified [22, 23]. Studies found that in patients with SARS-CoV-2 infection, pro-inflammatory cytokines were increased in plasma, but elevated IL-37 levels were accompanied by significantly lower levels of high-sensitivity C-reactive protein, IL-6 and IL-8, did not antagonize plasma Type I interferon levels, and a significant correlation was found between the parameters and the patient's clinical outcome and benign prognosis [24, 25]. Human IL-37b, the most complete and most reported isoform, can inhibit pro-inflammatory factors by acting as a negative regulatory signal, which plays a vital role in anti-inflammatory and anti-cancer activities [26, 27]. Thereby we predicted that inflammatory cytokines IL37b were also mainly derived from monocytes and inhibited inflammation in patients with DENV infection.

To verify the hypothesis that inflammatory cytokines IL37b were mainly derived from monocytes, explore the correlation of IL37b with IL-6, IL-17A, IFN-α, IL-1β, IL-10, and TNF-α, in our study, we collected blood samples from 53 cases of dengue fever from The Eighth People's Hospital of Dongguan City and 20 healthy volunteers’ blood were used as controls. PBMCs and Serums were isolated from each sample. PBMCs were used to do the flow cytometry analysis to explore the source of inflammatory cytokines. To determine inflammatory cytokines in serum and PBMCs, ELISA and RT-qPCR were performed (Fig. 1D).

### Elevated Plasma IL-37b in DF Patients are Mainly Derived from Monocytes but not Lymphocytes

**Fig. 2:**
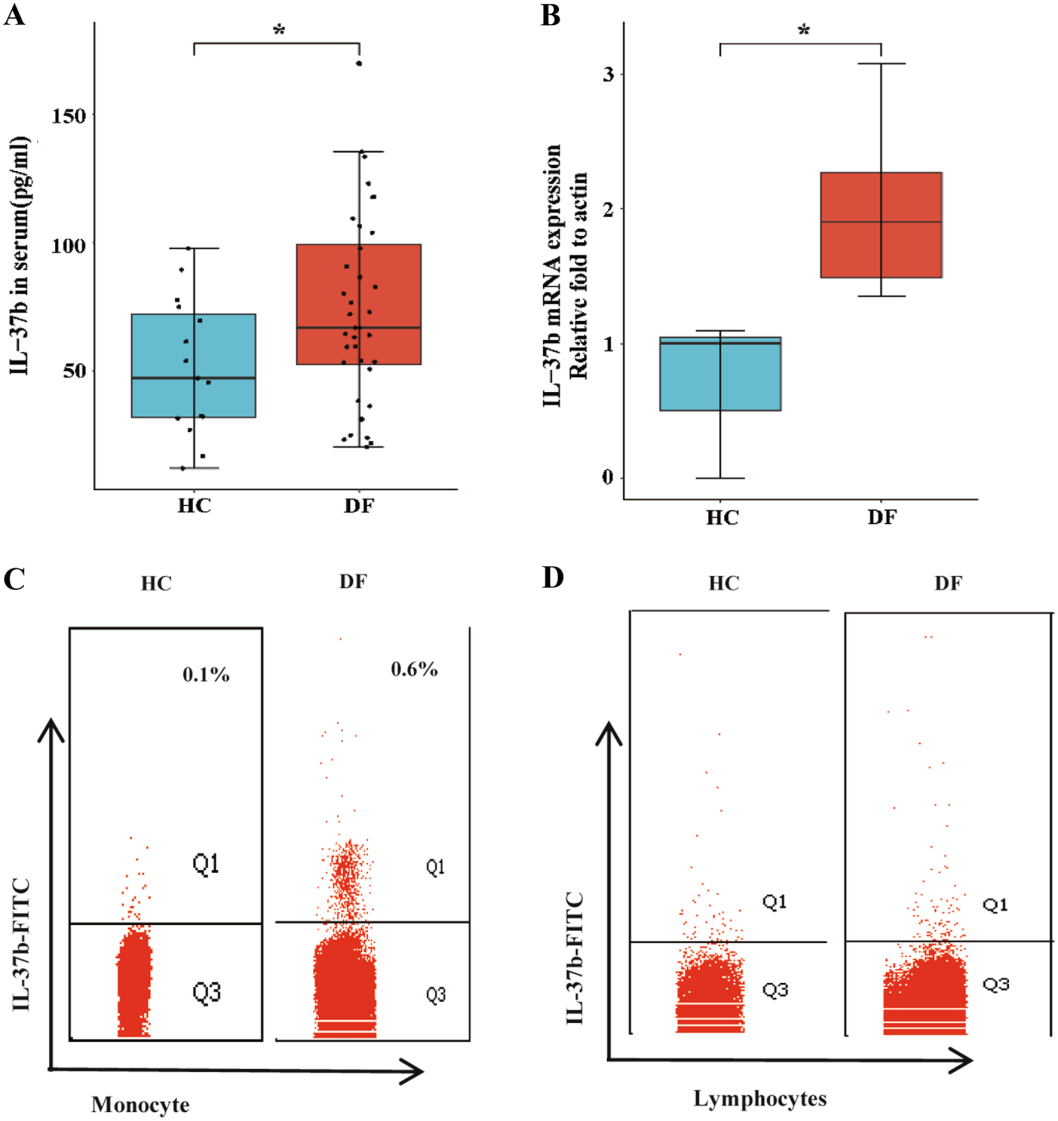
Coincident elevation serum IL-37b and monocytes producing IL-37b in patients with DF. **A** The IL-37b serum levels in DF patients were higher than that in HCs. **B** IL-37b mRNA levels in monocytes from DF patients was more higher compared to HCs. Gating strategy for monocytes and lymphocytes of PBMCs. IL-37b was mainly came from monocytes, but not lymphocytes **C**, **D**. The analysis was performed by Student’s *t* test or ANOVA by R package ggplot2. Data were shown as Mean ± SEM. *P < 0.05, **P < 0.01

An abnormal level of IL-37 is observed in patients with inflammatory and autoimmune diseases [23]. According to our previous studies we found that in tuberculosis patients, IL-37b was particularly sensitive to tuberculosis infection and maintained high levels of secretion in patients. In humans infected with dengue virus, however, the immune responses of IL-37b and its source of production are unknown. It was observed in this study that the levels of IL-37b in serum of DF patients were higher than HCs (Fig. 2A). As in human immune cells stimulated with LPS, monocytes provided the primary source of IL-37 [28], we examined the expression of IL-37b mRNA in monocytes of patients with DF using RT-qPCR. Monocytes from patients with DF expressed much more IL-37b mRNA than those from HCs (Fig. 2B). We then examined the IL-37b production of monocytes using intracellular cytokine staining (Fig. S1A, B). As expected, IL-37b was mainly produced by monocytes, but not lymphocytes (Fig. 2C, D). We also compared the level of IL-37 in lymphocytes and monocytes from HCs and DF patients, and discovered that, compared to HCs, DF monocytes contained higher levels of IL-37 (Fig. S1C), but the level of IL-37 in lymphocytes was not significantly different from that in healthy individuals (Fig. S1D). These results suggest monocytes may could serve as a source of elevated serum IL-37b in DF patients.

### Serum IL-37b and IL-37b-Producing Monocytes in DF Patients were Linked to IL-6, IL-10, and IFN-α

**Fig. 3:**
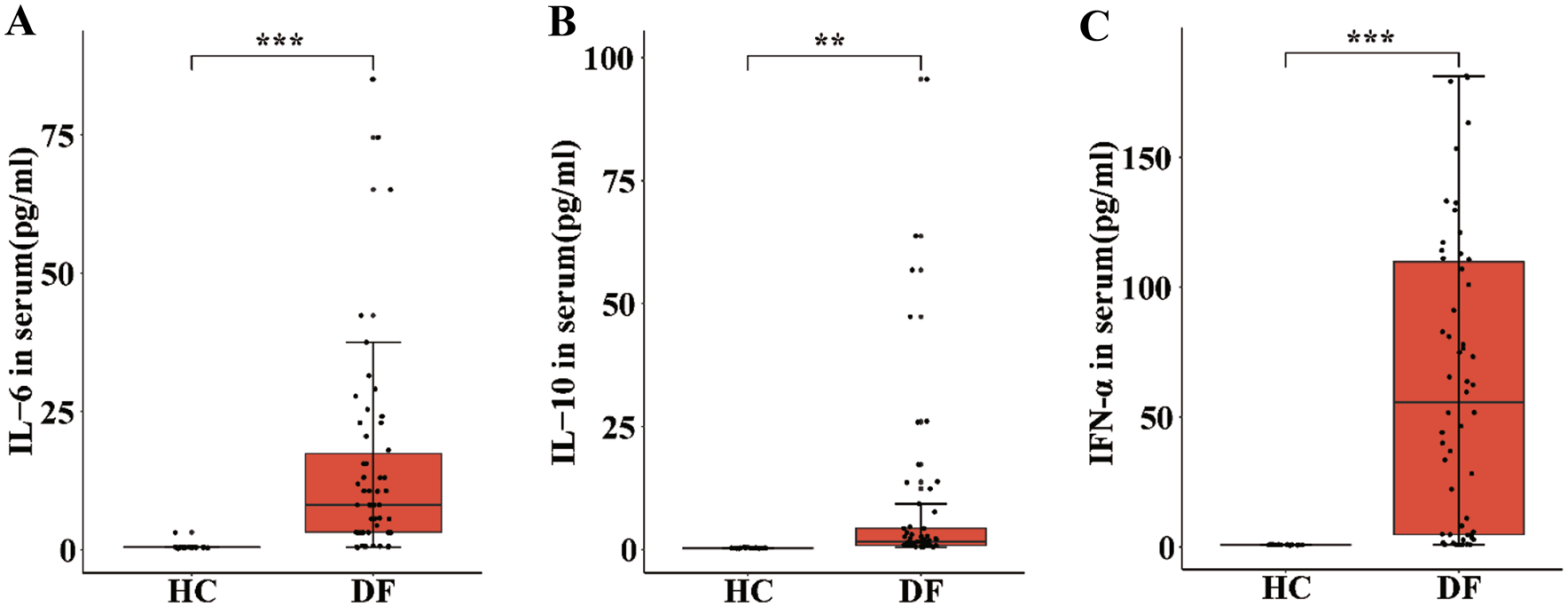
Increased inflammatory factors levels in serum with DF. Levels of IL-6, IL-10 and IFN-α were found to be higher in DF patients than in HCs (**A**–**C**). The analysis was performed by ANOVA or Student’s *t* test by R package ggplot2. Data were shown as Mean ± SEM. *P < 0.05, **P < 0.01

Several studies have found that IL-6, IL-17A, IFN-α, IL-1β, IL-10, and TNF-α play different roles in dengue disease [29–33], but we didn't know which factor play key roles or even correlated with IL-37b and other factors. Consequently, we measured the concentrations of these factors in DF patient and HCs sera. However, we failed to detect IL-1β, IL-17A, and TNF-α in plasma. Interestingly, serum levels of IL-6, IL-10, and IFN-α were found to be higher in DF patients than in HCs (Fig. 3A–C). We then examined whether these cytokines were implicated in the production of IL-37b in DF patients. The findings demonstrated that there was no correlation between IL-6, IL-10, and IFN-α expressions and IL-37b in serum (*P* > 0.05) (Fig. S2A–C).

**Fig. 4:**
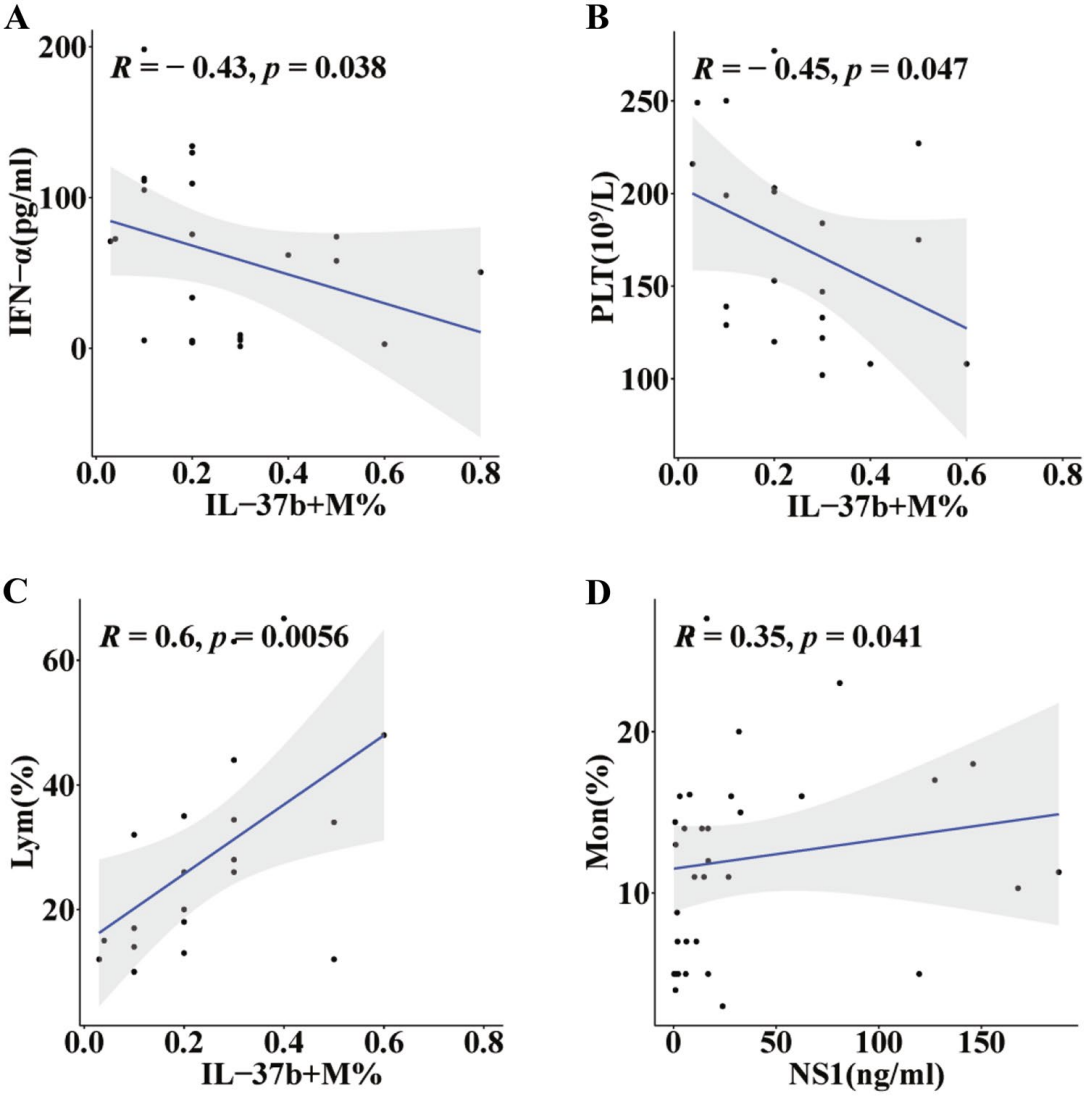
The correlation of IL-37b-producing monocytes and NS1 with laboratory values in patients with DF. **A** There was a significant negative correlation between levels of IFN-α and the percentage of monocytes producing IL-37b. **B** and **C** IL-37b-producing monocytes percentage were negatively correlated with platelet count, and positively correlated with the percentage of lymphocytes. **D** NS1 were positively correlated with monocytes percentage. Analyzed by Spearman´s correlation analysis statistically by R package ggpubr. *P* < 0.05 was considered as statistically significant

IFN-α and IL-1β are essential for the antiviral responses during dengue virus infection and have been previously displayed in DF patients with altered innate inflammatory factors [34], secretory IL-37b may play a greater regulatory role in the secretion of endogenous inflammatory factors in DF patients than previously thought. Thus, the dynamics of serum IL-6, IL-10 and IFN-α levels and IL-37b-producing monocytes in DF patients were investigated, and results showed that the level of IFN-α in serum was negatively correlated with IL-37b-producing monocytes (*P* < 0.05) (Fig. 4A), while the levels of IL-37b, IL-6 and IL-10 had no correlation with IL-37b-producing monocytes (*P* > 0.05) (Fig. S3A–C).

### No correlation Between NS1 and IL-37b, IL-6, IL-10, IFN-α Levels or IL-37b-Producing Monocytes

High serum NS1 levels, as a surrogate indicator of viral load have been found associated with disease severity [9]. In addition, NS1 antigen can directly activate mouse macrophages and human PBMCs via Toll-like receptor 4 (TLR4), inducing pro-inflammatory cytokine and chemokine production [35]. We tested the serum levels of NS1 in order to further investigate the relationship between IL-37b and dengue virus load, and the role of NS1 antigen levels on the regulation of inflammatory cytokines. It was found that serum NS1 levels were not related to IL-37b, IL-6, IL-10, IFN-α levels or IL-37b-producing monocytes (*P* > 0.05) (Fig. S4A–E).

### IL-37b-Producing Monocytes and NS1 are Correlated with Laboratory Values in DF Patients

Previous studies reported that the characteristic blood examination of DF patient was thrombocytopenia, which was led by platelet activation [35, 36]. In the context of DENV infection, activated and apoptotic platelets certainly play an important role in regulating monocytes' responses. Analyzing the relationship between IL-37b and immune cells could illuminate the effect of IL-37 on cell-mediated immunity in DF patients. Therefore, we investigated the correlation of IL-37b and IL-37b-producing monocytes with platelets, monocytes, and lymphocytes, and also investigated CRP levels in response to the impact of IL-37b on clinical laboratory values in DF patients. Surprisingly, we found that there was no correlation between serum IL-37 levels and platelet count, monocytes, lymphocytes, or CRP levels (Fig. S5A–D). In this study the percentage of monocytes producing IL-37 showed negative relation with platelet (Fig. 4B) and had a positive correlation with lymphocytes percentage (Fig. 4C), but not monocytes percentage or CRP levels (Fig. S5E–F). According to study, a sudden drop in platelet count can be a warning sign, as compared with patients with non-severe dengue, patients with severe dengue with a lower platelet count was significantly [37]. Furthermore, our study also found a positive relation between DENV NS1 levels and monocyte percentages in (Fig. 4D), but NS1 levels were not associated with platelet count, lymphocyte, and CRP levels. (Fig. S5G–I).

## Discussion

There are two main hypotheses regarding immune defense against dengue: antibody-dependent enhancement and cytokine storm [38]. The role of serum cytokines in the pathogenesis of dengue patients has become a hot topic of research in recent years. Additionally, there are a large number of studies analyzing the effects of cytokines associated with dengue patients, the changes of cytokines in serum of patients with different degrees of disease, and the interactions between cytokines. In chronic infectious diseases, inflammatory cytokine elevation is not strong enough to drive the immune effector cells to clear the virus, as the virus may induce a large number of anti-inflammatory cytokines to inhibit the inflammatory effect. We firstly re-analyzed a publicly available scRNA-seq dataset from the NIH GEO database (GSE154386). We found that common inflammatory cytokines IL10RA, IL17RA, IL17RE, IFNGR2, IL1B, and IL6 were mainly distributed in monocytes and mDCs. In our study, we confirmed that the IL-37b serum levels in DF patients were higher than that in HCs and IL37b were mainly derived from monocytes via ELISA and FCM, but RNA-seq dataset showed low abundance of IL-37 in monocytes and no statistical significance, preventing us from further analysis. Next-generation sequencing (NGS) technology relies on RT-qPCR and these experimental procedures may increase the error rate. NGS libraries for all types of applications contain biases that compromise the quality of NGS datasets and can lead to their erroneous interpretation. Moreover, RNA-seq protocols are technically more challenging than DNA-seq protocols and are often biased procedures. Interestingly, we found that the interacting genes of IL37 were not enriched in monocytes, but were highly expressed in NK cells and Vδ2 γδT cells, this may be related to intercellular communication, and as reported, IL-37 released from cells and then inhibits inflammation by binding to its cell surface receptor complex [39], such as IL1R8, IL-1 receptor family member expressed on the NK cells, which It may also lead to phenotypic changes and diminished function in canonical NK cells that have been suppressed by Tregs [28, 40].

In previous studies we found that IL-37b was particularly sensitive to tuberculosis infection and maintained high secretion levels in tuberculosis patients. IL-37b was determined as the entry point in DF [11]. IL-37, formerly referred to as IL-1F7, is a natural suppressor of innate inflammatory and immune responses [22], which has remarkable properties to suppress harmful inflammatory responses and prevent inflammation-mediated tissue damage [23]. Research into IL-37's sources, changes, and clinical implications in dengue patients will be beneficial to our understanding of the disease.

It was found that DF patients had higher serum IL-37b levels and monocytes that produced IL-37b compared to healthy participants. It has been shown that the monocytes, DCs and T cells are the origins of IL-37 among immune cells [23], and PBMCs immunocytochemical staining revealed that IL-37b protein is mainly presented in the cytoplasm of monocytes [41]. To substantiate where did the elevated plasma IL-37b come from, we firstly detected the expression of IL-37b mRNA in monocytes of DF patients by RT-qPCR, and then measured the distribution of IL-37b in PBMCs by intracellular factor staining. This experimental design is similar to our earlier study [11].

Our results imply that dengue also elicits elevations of several immune factors. High expression of IL-6, IL-10, and IFN-α in DF patients was consistent with previous studies which showed that infection with dengue virus leads to elevated levels of these cytokines which are significantly increased in plasma of dengue patients [15, 20, 42, 43]. This result is also consistent with our scRNA-seq results which indicate that DENV activates the innate immune system, resulting in the release of multifunctional cytokines. But unexpectedly, we could not detect IL-1β, IL-17A or TNF-α in dengue samples because of off-target. In addition, results revealed a negative correlation between IL-37b + monocytes and IFN-α expression. But IL-37b level showed no correlation with IFN-α expression. As is known to all, dengue infection can trigger the host to produce IFN-α, which is one of the most powerful and widespread antiviral agents [33]. From previous results, it is clear that Dengue patients had higher serum levels of IL-37b as well as monocytes that produced IL-37b compared to healthy controls, and elevated serum IL-37 is mainly derived from IL-37-producing monocytes, but to our surprise, there is no correlation between serum IL-37b and IL-37b-producing monocytes. This may be because the sample size is so small that the correlation could not support the previous conclusion that elevated serum IL-37 is mainly derived from IL-37-producing monocytes. Notably, IL-37b level and percentage of IL-37b^+^ monocytes have no relation to IL-6, IL-10. Sai et al. found that IL-37 acted as a negative feedback suppressor of the inflammatory response, which was not dependent on anti-inflammatory cytokines such as IL-10 [22], which was consistent with our results.

Dengue NS1 antigenemia has been utilized as a surrogate indicator of viral load [44]. Results indicated that no correlation between serum NS1 levels and IL-37b, IL-6, IL-10, IFN-α levels and IL-37b-producing monocytes. This was not surprising for IFN-α levels as previous studies have shown that dengue virus infection can suppress the production of IFN-α to promote the virus’s evasion from immune responses.

IL-1β and IL-6 are both associated with the severity of dengue fever [33, 45], promoting the activation of IL-1β and leading to tissue damage and vascular leakage [31]. IL-6 plays a crucial role in coagulation dysfunction and could lead to plasma leakage and bleeding [46]. Unfortunately, due to the insufficient number of samples in this study, the relationship between these inflammatory factors and L-37b level or percentage of IL-37b + monocytes levels could not be further elucidated, which needs to be explored further. According to some studies, IL-1 β IL-6, and IFN-α levels were significantly higher in patients with severe dengue compared to those with mild dengue, which were considered as potential predictors of severe dengue infection [45, 47]. In addition, studies have also shown that there is no significant correlation between viral load and dengue severity [44]. However, our current study fails to classify dengue patients by severity, and the relationship between these inflammatory factors, viral load and dengue severity has not been clarified, which also needs to be further explored.

Platelet activation and thrombocytopenia are thought to be characteristic of dengue infection which can lead to severe dengue fever, DHF, and DSS [35]. Because abnormal cytokines production is also proposed to be one of the mechanisms of vascular leakage in DHF/DSS, we analyzed the correlation of serum IL-37b and IL-37b-producing monocytes with platelets. And we found that platelets counts were negatively associated with monocytes producing IL-37b, whereas no association with IL-37b level. Platelets are reported to aggregate with monocytes during dengue infection, causing specific cytokine responses leading to thrombocytopenia and increased vascular permeability in dengue patients [48, 49]. Therefore, it is reasonable to suspect that high levels of IL-37b-producing monocytes and low platelets in patients are partially due to increased platelet-monocytes aggregation. In addition, we found that the percentage of lymphocytes was positively associated with IL-37b-producing monocytes, and monocytes was positively associated with the level of NS1. Further research is needed to understand whether this is related to changes in immune cells over time during dengue virus infection.

It has been demonstrated that higher CRP levels indicate more severe inflammation and tissue damage; CRP levels are significantly higher in patients with acute phase, high viral load, and secondary infection [50]. Our study showed that, there was no correlation between CRP and the level of IL-37, IL-37b-producing monocytes or NS1 level, so we assumed that the patients are neither in the acute phase nor in the secondary infection stage. Whether IL-37 has a regulatory effect on CRP levels in dengue patient needs to be further investigated.

## Conclusion

Our data propose that IL-37b may be associated with dengue fever, but the biggest flaw of our experiment is lacking patients with DHF or DSS, resulting in we could not establish a connection between IL-37b and the clinical severity of the infection. In addition, there is some ambiguity regarding the expression of IL-37b in dengue patients. If, and to what extent IL-37b can judge the seriousness of dengue fever needs to be further explored.

## Supplementary Information

Below is the link to the electronic supplementary material.

**Fig. S1** Differences in the content of IL-37b in PBMCs of monocytes and lymphocytes in HCs and DF patients.** A** and** B** An intracellular ctokine staining method was to examine IL-37b in PBMCs by an IL-37b mAb.** C** IL-37b level in monocytes of DF patients was higher compared to HCs.** D** Levels of IL-37b in lymphocytes was not significantly different from that in normal people. The analysis was performed by Student’s t-test or ANOVA by R package ggplot2. Data were shown as Mean±SEM. *P < 0.05, **P < 0.01. (TIF 27354 KB)

**Fig. S2** Serum IL-37b levels showed no correlation with IL-6, IL-10 and IFN-α in DF patients (**A**–**C**). Analyzed by Spearman´s correlation analysis statistically by R package ggpubr. *P* < 0.05 was considered as statistically significant. (TIF 27370 KB)

**Fig. S3** The correlation of IL-37b-producing monocytes with IL-6, IL-10 in DF patients. Serum IL-37, IL-6 and IL-10 levels were not found to be correlated with the percentage of IL-37b-producing monocytes. Analyzed by Spearman´s correlation analysis statistically by R package ggpubr. *P* < 0.05 was considered as statistically significant (TIF 27659 KB)

**Fig. S4** No correlation of NS1 antigen levels with IL-37b, IL-6, IL-10, IFN-α levels or percentage of IL-37b-producing monocytes in serum of DF (**A**–**E**). Analyzed by Spearman´s correlation analysis statistically by R package ggpubr. *P* < 0.05 was considered as statistically significant (TIF 27896 KB)

**Fig. S5** The correlation of serum IL-37b, IL-37b-producing monocytes and NS1 with laboratory values in patients with DF. Serum IL-37b levels were not correlated with platelet count, lymphocytes, monocytes percentage or CRP levels (**A**–**D**). IL-37b-producing monocytes percentage were not correlated with the percentage of monocytes (**E**) or CRP levels (**F**). NS1 were not related with platelet, lymphocytes percentage or CRP levels (**G**–**I**). Analyzed by Spearman´s correlation analysis statistically by R package ggpubr. *P* < 0.05 was considered as statistically significant. (TIF 27408 KB)

## Data Availability

All data used for the analysis in this study are available upon reasonable request to the corresponding authors.
