# Supplementary figures and images for: Elevated Interleukin-37 Associated with Dengue Viral Load in Patients with Dengue Fever
Source: Curr Microbiol. 2023 Apr 6;80(5):171. doi: 10.1007/s00284-023-03239-7 (PMC10079153; doi:10.1007/s00284-023-03239-7)

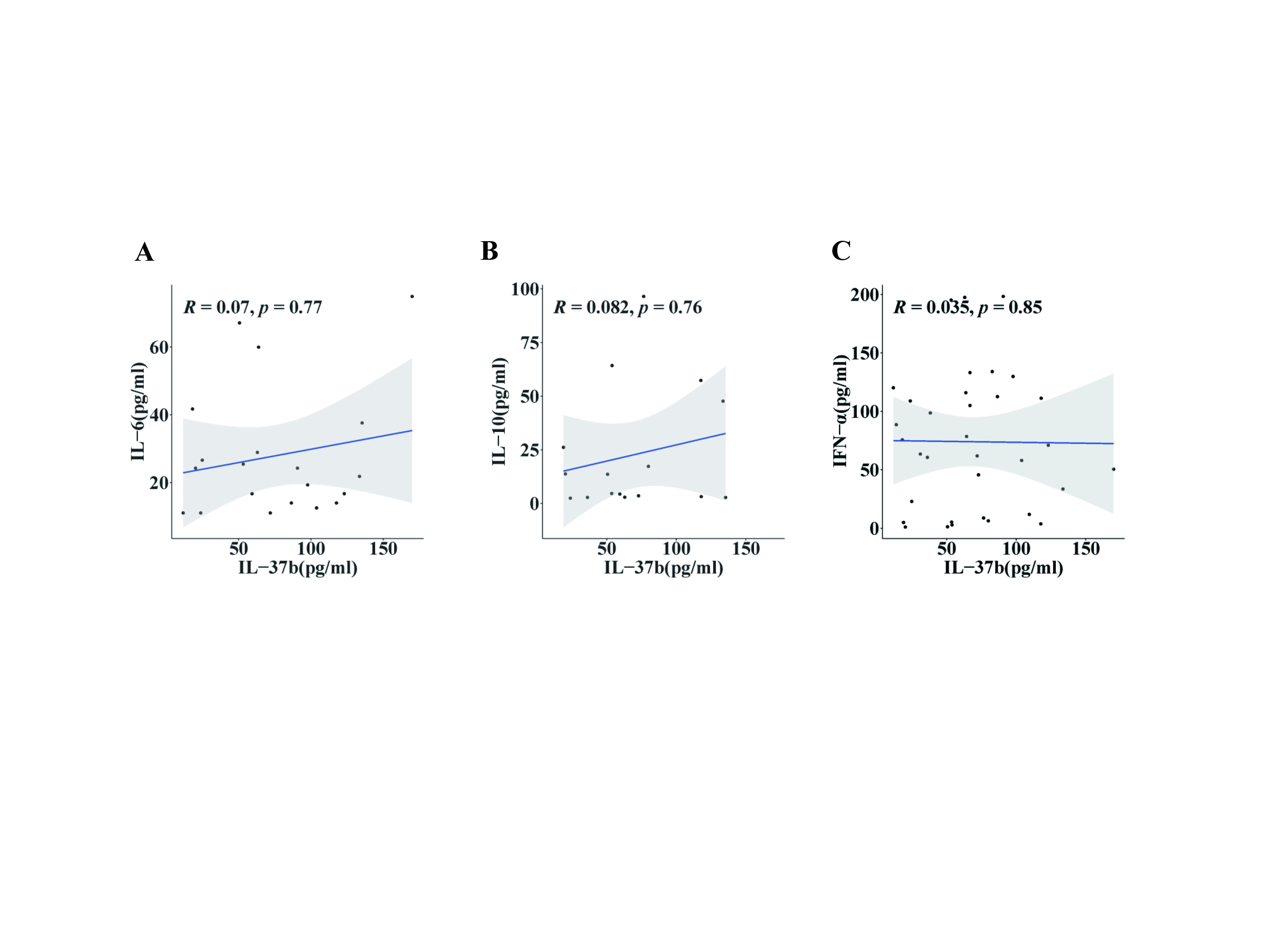

Supplement: Supplementary file 1 — Fig. S1 Differences in the content of IL-37b in PBMCs of monocytes and lymphocytes in HCs and DF patients. A and B An intracellular ctokine staining method was to examine IL-37b in PBMCs by an IL-37b mAb. C IL-37b level in monocytes of DF patients was higher compared to HCs. D Levels of IL-37b in lymphocytes was not significantly different from that in normal people. The analysis was performed by Student’s t-test or ANOVA by R package ggplot2. Data were shown as Mean±SEM. *P < 0.05, **P < 0.01. (TIF 27354 KB) [file 284_2023_3239_MOESM1_ESM.tif]

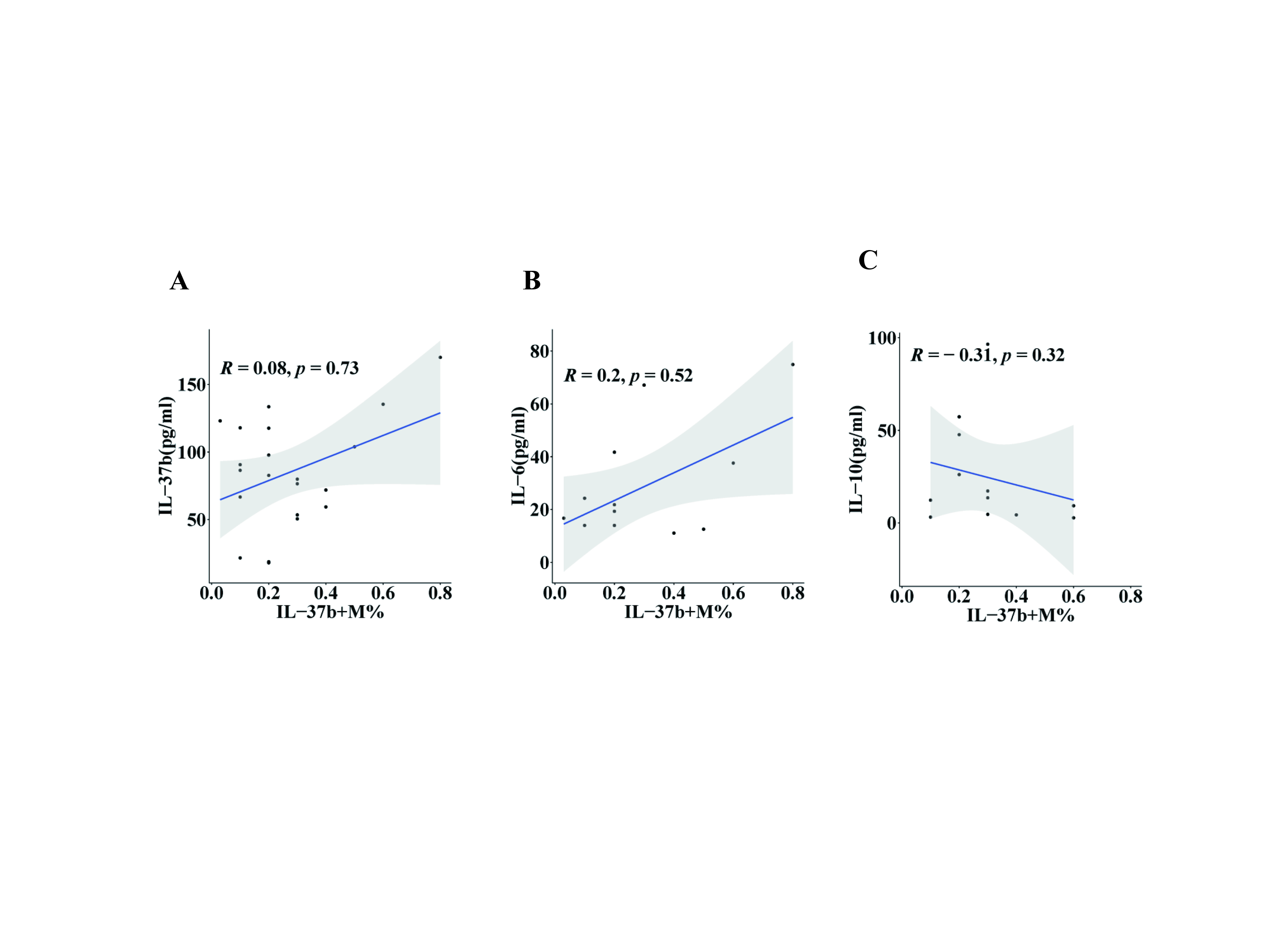

Supplement: Supplementary file 2 — Fig. S2 Serum IL-37b levels showed no correlation with IL-6, IL-10 and IFN-α in DF patients (A–C). Analyzed by Spearman´s correlation analysis statistically by R package ggpubr. P < 0.05 was considered as statistically significant. (TIF 27370 KB) [file 284_2023_3239_MOESM2_ESM.tif]

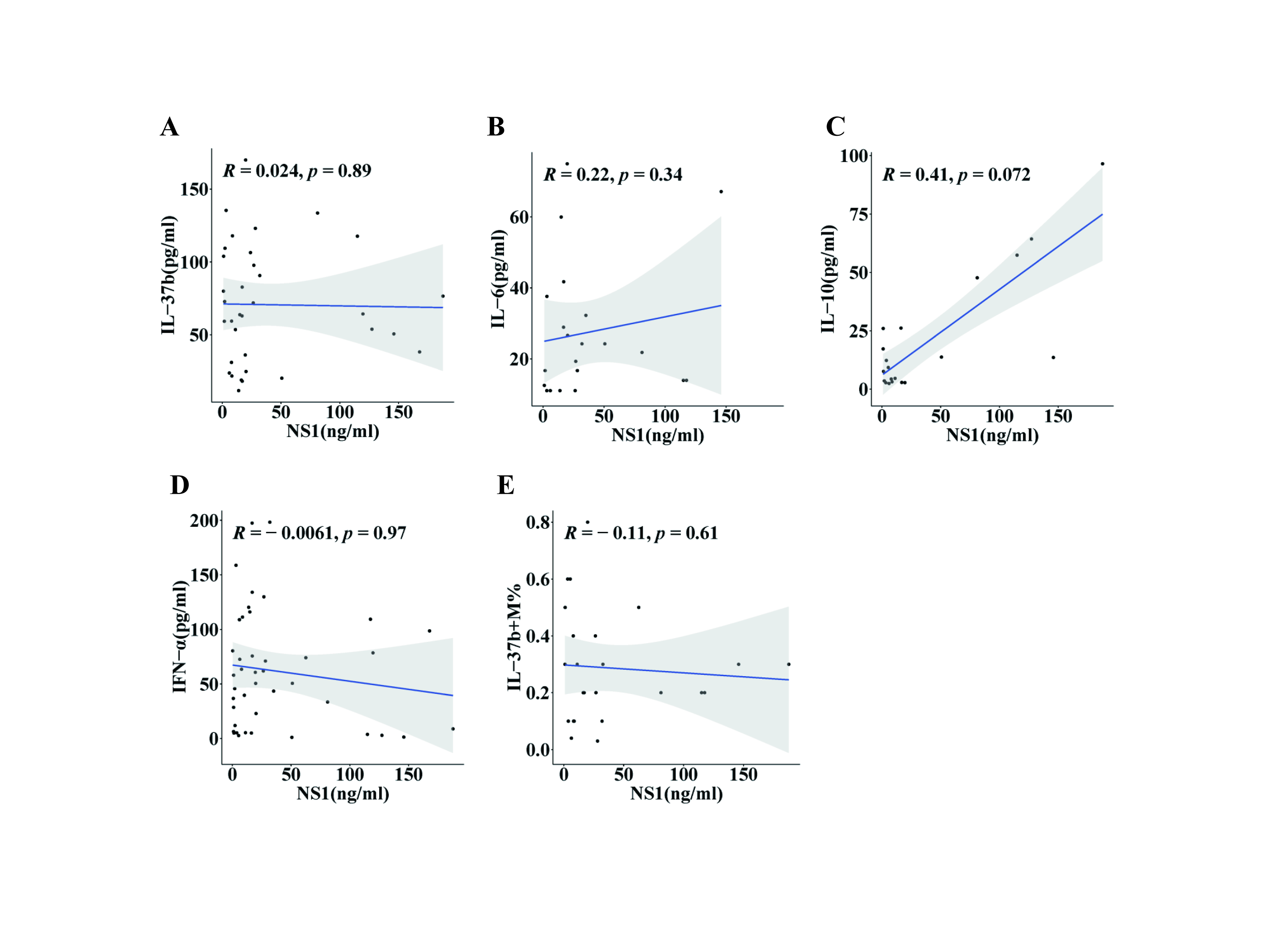

Supplement: Supplementary file 3 — Fig. S3 The correlation of IL-37b-producing monocytes with IL-6, IL-10 in DF patients. Serum IL-37, IL-6 and IL-10 levels were not found to be correlated with the percentage of IL-37b-producing monocytes. Analyzed by Spearman´s correlation analysis statistically by R package ggpubr. P < 0.05 was considered as statistically significant (TIF 27659 KB) [file 284_2023_3239_MOESM3_ESM.tif]

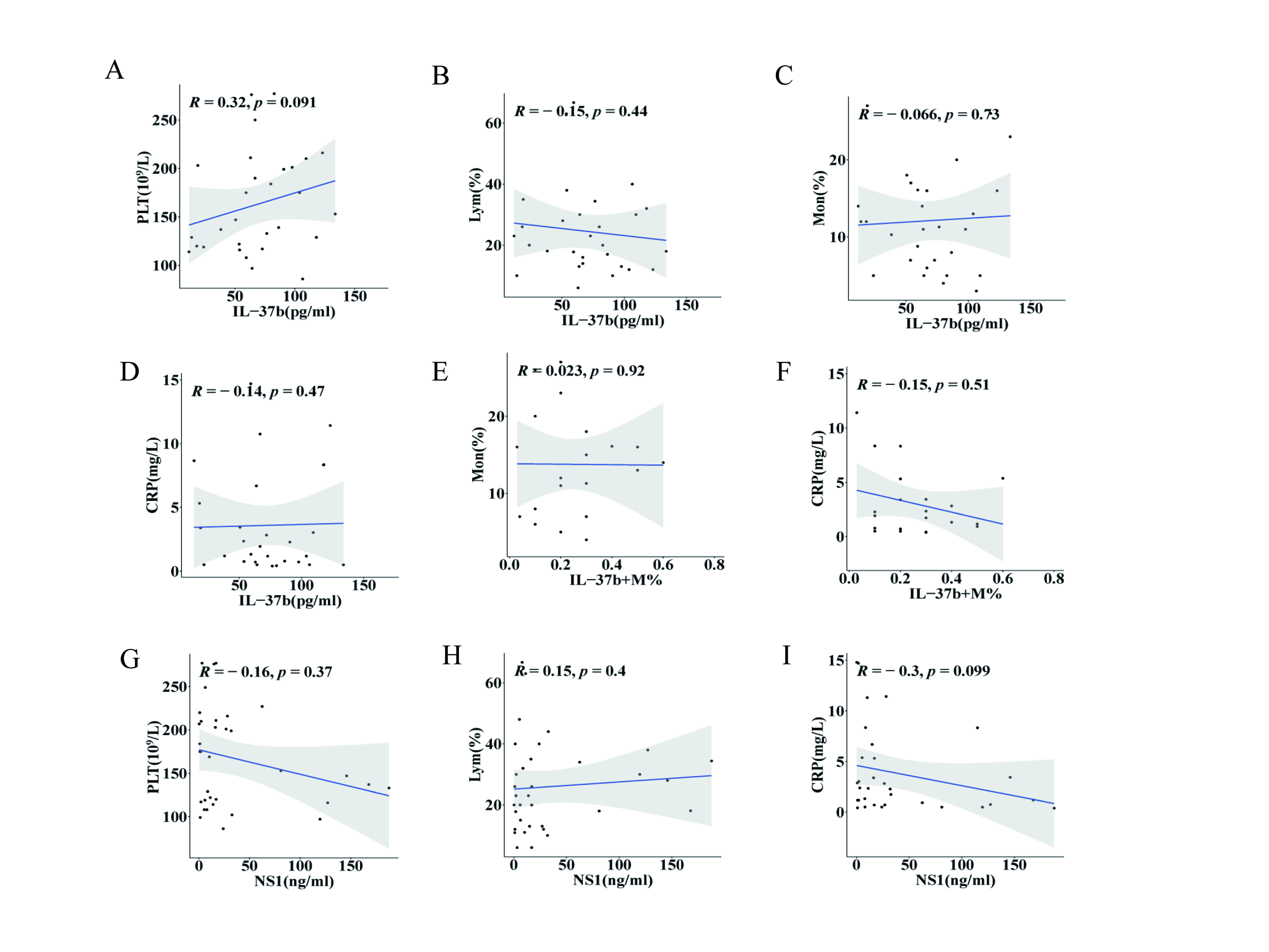

Supplement: Supplementary file 4 — Fig. S4 No correlation of NS1 antigen levels with IL-37b, IL-6, IL-10, IFN-α levels or percentage of IL-37b-producing monocytes in serum of DF (A–E). Analyzed by Spearman´s correlation analysis statistically by R package ggpubr. P < 0.05 was considered as statistically significant (TIF 27896 KB) [file 284_2023_3239_MOESM4_ESM.tif]

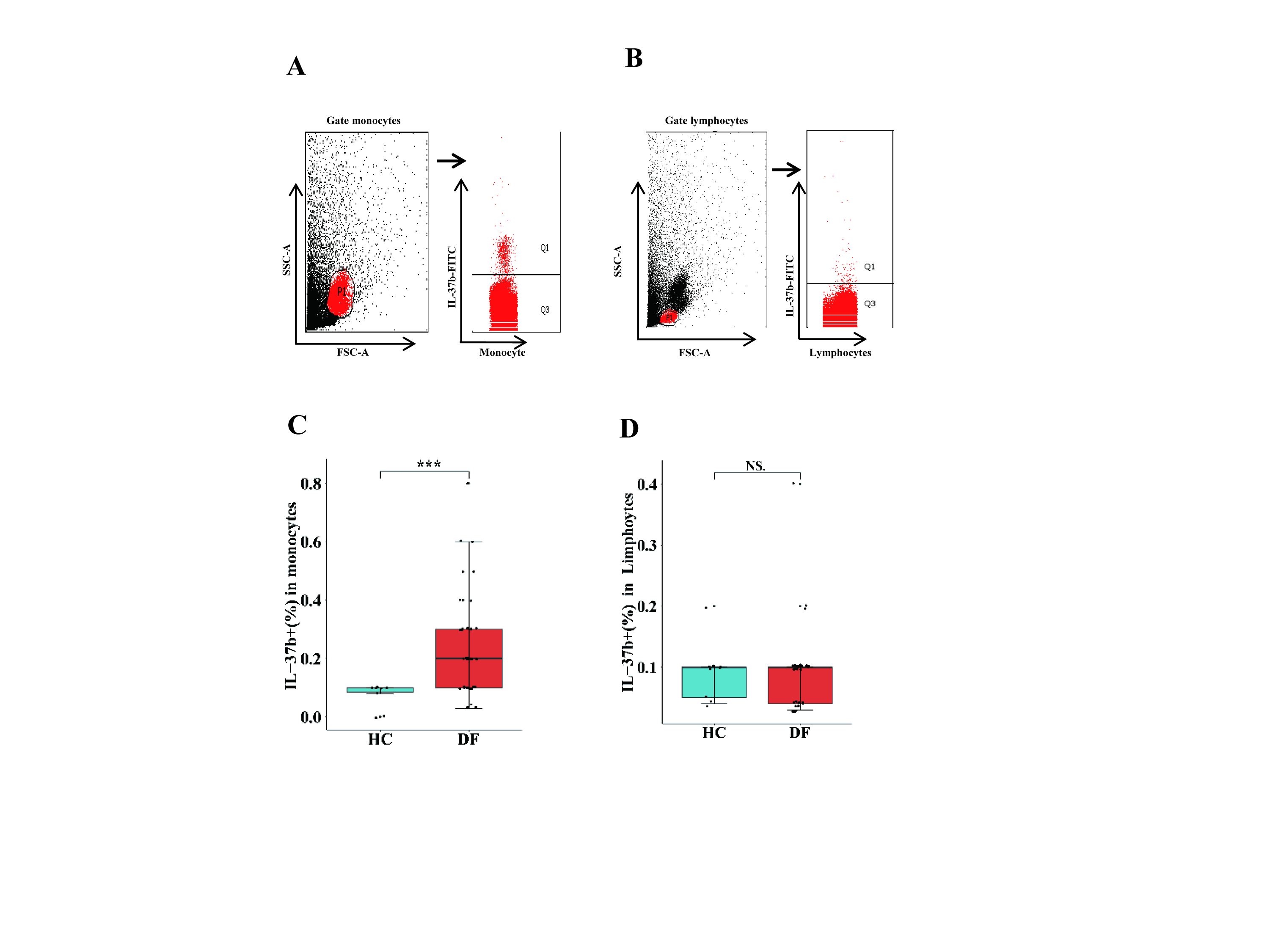

Supplement: Supplementary file 5 — Fig. S5 The correlation of serum IL-37b, IL-37b-producing monocytes and NS1 with laboratory values in patients with DF. Serum IL-37b levels were not correlated with platelet count, lymphocytes, monocytes percentage or CRP levels (A–D). IL-37b-producing monocytes percentage were not correlated with the percentage of monocytes (E) or CRP levels (F). NS1 were not related with platelet, lymphocytes percentage or CRP levels (G–I). Analyzed by Spearman´s correlation analysis statistically by R package ggpubr. P < 0.05 was considered as statistically significant. (TIF 27408 KB) [file 284_2023_3239_MOESM5_ESM.tif]
